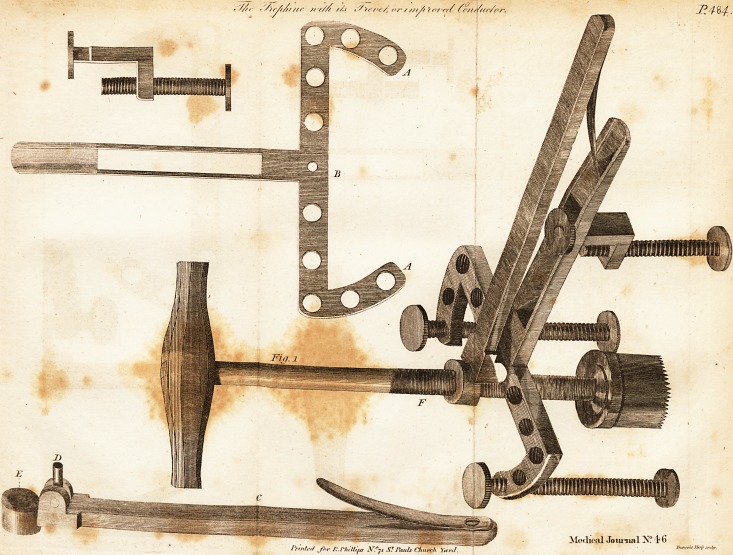# Description of an Apparatus Attached to the Trephine, in the Operation of Trepaning, by Which It Is Effectually Prevented from Slipping Suddenly down upon the Brain

**Published:** 1802-12-01

**Authors:** John Savigny

**Affiliations:** King Street


### Medical and Phyfical Journal.

VOL. VIII.] DECEMBER 1, 1802. [NO. XLVI.

*Printed for R. PHILLIPS, by W. Thorne, Red Lion Court, Fleet Street, London.*

### *To the Editors of the Medical and Phyfical Journal.*

GENTLEMEN,

HAVING been employed by Mr. William Jardine, to conftruct feveral new invented Inftruments of Surgery, and fortunate enough to have acquitted myfelf much to his fatisfaction in their execution, he is defirous (as a teftimony of approbation towards me, and from a conviction of the utility and importance of his productions) to prefent the defigns and defcriptions of them to the public; and has commiffioned me not only to fuperintend the engraving of the plates, and arrangement of the defcriptions, but to entreat that they may be favoured with a place in your extenfive and moft ufeful Publication. He has alfo favoured me with a Letter, which, in ftrict propriety on *my part*, fhould perhaps have been fuppreffed, but which he has exprefsly defired may accompany the infertion as a neceffary and proper introduction. In complying with thefe his inftructions and wifhes, I have availed myfelf of the opportunity afforded, of introducing a reprefentation of an improved method of conftructing the feton needle, an alteration in the form of this inftrument having become indifpenfable, when ufed with a tape prepared with a folution of the elaftic gum, according to a late ingenious and elegant improvement: As the nature of this tape would not permit it to adapt itfelf in folding or doubling when paffed through the eye of the needle, fo compactly as the common fkein, inftead of the eye, I flit the fteel for about three-quarters of an inch, in the flat direction of the needle, and adjuft the two parts fo as to form a kind of fpring forceps, holding the tape with fufficient firmnefs to draw it through the flefh perfectly fmoothly, and without the leaft obftruction.

In the hope that thefe Communications may be deemed worthy of appearing in your valuable Collection,

I am, &c.

*King Street, Nov.* 13, 1802. JOHN SAVIGNY.

### Trephine and Trevet.

In prefenting the following inventions to the public, it is not improbable but the common objection againft increafing the number of inftruments, in any, but more efpecially the furgical art, will be incurred. This objection muft, in a certain degree be admitted, where the laft productions are not better than the firft, or do not fuperfede their ufe. Experience alone muft decide whether or not the defigns now fubmitted will fall under this cenfurable defcription.

It has frequently, and with much reafon been obferved, that the principles of new invented or improved inftruments (if at all complicated in their appearance) are only or at leaft beft underftood by the inventors or improvers themfelves: To obviate this remark, it is much to be wifhed, that young furgeons were more generally inftructed in the ufe and management of inftruments, that by attention and *practical* application of them on dead fubjects, or a variety of other appropriate fubftances, they might acquire that mechanical adroitnefs in their actual ufe, in which, it is to be lamented, they are fo frequently deficient, and a more ready comprehenfion of the advantages of improvements in their conftruction, which it is acknowledged and *felt*, is fo very difficult to convey by the beft engraved reprefentations or the moft careful and ftudied defcriptions. Apprentices in all mechanical profeffions are ufually firft accuftomed to the ftructure and proper application of their tools, before they are allowed or expected to finifh any of their refpective works; why Gentlemen, intended for the nobleft of all profeffions, fhould not be initiated with equal care in knowledge fo far more important, has long been matter o aftonifhment and regret, fince it may fairly be prefumed, that i they were, or if their own natural talents were induftrioufly exerted towards the attainment of this moft effential branch of profeffional ability, the advantages to the fcience, and to mankind in general, would be as happy as incalculably great.

*Description of an Apparatus attached to the Trephine, in the Operation of Trepaning, by which it is effectually prevented from slipping suddenly down upon the Brain.*

In this contrivance, the circular cylindrical faw in common ufe is fufpended by its fhank (cut with a fcrew thread or worm of extremeft finenefs) fcrewed through the extremity of a lever, moveable by a pivot upon the fuperior part of a trevet, or triangular reft. This trevet is adjufted to any part of the head, and the faw it fufpends, brought at the fame time upon the portion to be perforated, by lengthening or fhortening the legs or branches of the trevet, which, for thefe purpofes, are furnifhed with fcrew threads, and pafs through the upper frame or top.

In

*The improved Lenticular, Raspatory, Tooth punch, Extractor for the Œsophagus, and Seton Needle.*

Medical Journal No. 46

Francois Herff sculp.

Printed for R Phillips No. 71 St. Pauls Church Yard.

In the fame manner, by varying the length of either leg, the fide of the faw is raifed or depreffed during the operation, as the inequality of the bone may require. The fine fcrew upon the fhank is alfo of further ufe in placing the faw, with greater exactnefs, upon the part intended, and allowing it to enter as deep as neceffary, while it prevents it, in its working, from paffing too fuddenly through the perforation, and thereby injuring the brain.

The fcrew upon the fhank fhould be of fo fine a thread, that though conftantly turned, it fhould not pafs fafter through the lever than the faw does through the bone.

The thread of the fcrews upon the legs of the trevet fhould be coarfe, in order to regulate its pofition or neceffary variations with more celerity; and the legs fhould, in adjufting it, be placed as nearly as the condition of the part to be operated upon will admit, at equal diftances from each other.

Before applying the faw, an impreffion fhould be made in the bone deep enough to confine the faw in its place while working; this may be moft readily effected by the new inftrument invented for that purpofe, and reprefented by fig. 2. of the next plate.

In working the inftrument, the operator with his left hand grafps the lever and one of the legs of the trevet together, and with his right, directs the handle of the faw; if the fhank of which fhould appear to pafs quicker through the lever than the teeth do through the bone, (an occurrence the operator will readily be fenfible of by the irregular motion or unfteadinefs of the lever) he muft occafionally give the handle a flight turn in the oppofite direction; but this is an inconvenience which from the finenefs of the fcrew upon the fhank, and the manner of holding the inftrument in the left hand with proper management, will feldom or never occur.

It has been obferved by a celebrated practitioner, (to whom this invention has been fubmitted) that " although it will, with much certainty, anfwer its defign of protecting the brain from injury, by the trephine falling in too fuddenly upon it; it will require a greater length of time in working, than either the trepan or trephine, and that any perfon, accuftomed to operate either upon the dead or living body, may eafily avoid the inconvenience which this inftrument is intended to obviate, and that no furgeon ought to operate who has not had many opportunities of diffecting, and performing operations upon dead fubjects."

Although, to practitioners of experience, fuch a guard may be unneceffary, if it but enable the timid and inexperienced operator to proceed in fuch a nice and hazardous operation with greater expedition and fafety, than from a want of confidence

 in in his own dexterity he otherwife would, one very important end is gained by the improvement.

If no furgeons were allowed to perform this operation but thofe who have had fuch opportunities of diffecting, &c. what muft become of the greateft part of mankind, liable to fuch accidents, who are obliged to take fuch affiftance as they can get; for, comparatively fpeaking, very few furgeons indeed have had the opportunities mentioned, and yet amongft fuch, are found very clever men, who have performed the operation in queftion, very dexteroufly and very fuccefsfully.

The fame trevet, with a little alteration or addition, may anfwer the purpofe of a Levator, by fubftituting a fecond lever, with a coarfer fcrew at its extremity; and inftead of the trephine, another inftrument to pafs through it, in the famè manner, with its inferior end properly conftructed for the purpofe. In this cafe, the handle of the inftrument, in its ufe, muft be turned the contrary way to *that* when the trephine is employed in making the perforation.

Fig. 1. Reprefents the trevet, with the faw fufpended, ready to be applied to the part to be perforated. A. The top of the trevet, with its various holes for receiving the feet, and occafionally fhifting them. B The hole to receive the pivot of the lever. C. The lever feparated from the trevet. D. The pivot which connects the lever to the top of the trevet. E. The hole in the end of the lever through which the faw paffes. F. The fine fcerw upon the fhank of the trephine.

### Improved Lenticular.

The Lenticular is an inftrument, apparently better adapted to its intent, than experience can allow to be the cafe. Were the bones to be perforated of a fofter nature than they are, it might probably fucceed perfectly well; but to cut off all the pointed and unequal parts neceffarily remaining after the perforation of fo hard a fubftance as the cranium, under all the various circumftances of fo delicate an operation, requires an inftrument of a very different conftruction to the common lenticular.

The inftrument hitherto employed for this purpofe, requires more force, or at leaft more fteady application, than can be exerted by the moft expert operator in turning it, fo as to remove with indifpenfable fmoothnefs the pointed and hard fplinters, fo clofe to fuch a delicate and irritable membrane, while the fragments which the cup was intended to receive are, by the unavoidably unequal and unfteady motion of the hand, very liable to be thrown out of it.

The invention recommended as its fubftitute, is formed of two fegments of a circle of about the fame depth as the common faw, one of them with a cutting point, or angle, fomewhat prominent. They are fixed upon the ftem of a handle, at

*The Trephine with its Trevet, or improved Conductor.*

*Printed for R. Phillips N.º 71 S.t Pauls Church Yard.*

*Francois Heriy sculp.*

at fuch a diftance from each other, that when compreffed within a circle, they act with fome degree of force againft its internal edges.

It refembles, in fome refpects, the circular faw with two parts of its circle removed; the cup to receive the fplinters is fixed upon the inferior extremity of the cutting part.

It is adjufted to circles of different diameters, by a fpring connected with the cutting part. The fkull being perforated, on gently preffing with the thumb and fingers of the hand that holds the inftrument, the fides or fegments, they are made to approach each other, and are thus introduced into the circle with the projecting part of the cup under the part of the bone, which the operator may think moft proper; the depth of the oppofing fegment is made variable (by fliding it upon the ftem of the handle) as the inequality of the part, or its contiguity to the brain, may require. The fpring giving the fegments a diftending power, they confequently prefs againft the interior part of the circle; the operator has therefore nothing more to attend to than to turn the inftrument round, drawing the projecting cup gently up againft the under part of the cranium, when, with one revolution, all the prominent and ragged parts are removed with fmoothnefs and delicacy. On extracting the inftrument, the fame mode of preffing its fides or fegments towards each other muft be obferved, as on its introduction.

Fig. 1. A View of the improved Lenticular. A. The fegment with the cutting part fomewhat prominent at B. C. The receiving cup. D. The projecting part of the cup. E. The fliding fegment oppofing the cutter, adjufted by the fcrew, F. G. The handle. H. The ftem of the handle. I. A fpring to regulate the diameter of the inftrument. K. A milled head fcrew, by which the ftrength of the fpring is increafed or diminifhed.

### Improved Raspatory.

Although in the operation of the trepan, many furgeons have now laid the rafpatory afide as unneceffary, or ill calculated for its purpofe, it may certainly be more eafily and more expeditioufly performed, when, before the application of the faw, the pericranium is removed; but it muft be admitted very difficult to fcrape off juft fo much of the membrane as is merely neceffary for perforating the fkull, without an inftrument, with which we may defcribe a circle of equal diameter with the faw intended to be ufed. The rafpatory in common ufe is by no means adapted to this end; either *more* of the bone will be denuded by it than is neceffary, or the faw muft be impeded in its progrefs by *too little* of the membrane being removed.

The defigned inftrument (fig. 2) will not only readily remove the portion of membrane neceffary for the admiffion of the the ſaw, but is attended with this further advantage, that it will make a circular impreſſion of depth ſufficient to confine it in its place, and of courſe, to retain it perfectly ſteady in the beginning of the perforation (a part of the operation I have ſometimes ſeen attended with much difficulty) ſo as to ſuperſede the neceſſity of the centre pin of the trephine; or ſhould the operator wiſh in particular caſes to perforate *part* of the circle only, to aſcertain the thickneſs of the cranium, or any other deſired information, he may, by this inſtrument, ſatisfy himſelf, without retarding the operation.

This invention has been proved, on trial, to make its way into the cranium much faſter than the ſaw, and, for expedition, may be uſed conjointly with it, beginning the operation with the former, and concluding it with the latter.

The inſtrument nearly reſembles a cooper's marking iron, and is conſtructed to form circles of different diameters, by means of an adjuſting ſlide, to which the cutter or blade is fixed. The centre pin is formed as a ſcrew nearly its whole length, and riſes into the handle, as the cutter ſinks into the bone, thus rendering the ſide of the circle more perpendicular than it otherwiſe would have been, and, in conſequence, the ſaw more ſteady in its future progreſs. It is furniſhed with two blades, one ſcooped, the other plain. They ſucceed equally in perforating the bone, but are attended with this difference, the ſcooped blade, according to the original idea, cuts out the membrane, and ſinks into the bone, while the plain one, though it cuts faſter, cannot anſwer all the purpoſes of the raſpatory.

Fig. 2. Repreſents the improved Raſpatory. A. The handle. B. The centre ſcrew. C. The adjuſting ſlide, to adapt the inſtrument to circles of various diameters. D. The ſcrew to fix the adjuſting ſlide. E. The ſcooped blade fixed. F. The cutting blade. G. The ſcrew for fixing or removing the cutter.

The method of adapting the inſtrument to ſaws of different diameters, and the addition of the plain cutter, are improvements by Mr. Savigny.

### Instrument for extracting Stumps. (Fig. 3.)

The gum having been properly ſeparated, the operator, with his right hand, takes a firm hold of the handle of the inſtrument and preſſes down the point of the blade, (the ſerrated edge undermoſt) between the ſoundeſt ſide of the ſtump and the adjoining tooth, if he can there introduce the inſtrument.

The point being, in the opinion of the operator, ſufficiently depreſſed, he then turns the ſerrated edge upwards, keeping it cloſely againſt the ſide of the ſtump, and deſcribing in its action nearly a ſemicircle, ſometimes obtaining a fulcrum from the adjoining tooth, if found, by the preſſure of the back of the inſtrument againſt it.

### Instrument for Œsophagus Cases.

As many ſharp pointed ſubſtances, ſuch as fiſh-bones, &c. apt to ſtick in the throat, cannot, without danger of wounding the œſophagus, be forced down into the ſtomach, an inſtrument is propoſed for *extracting* ſuch ſubſtances, which I preſume will be found to anſwer its purpoſe better than any thing of the kind I have yet ſeen, and is ſo very eaſy in its application, that *any perſon*, with a moderate dexterity, to whom its principles have been once explained, need not heſitate, in a caſe of extremity or diſtreſs, to apply it. Hence, peculiarly ſerviceable, when in ſuch accidents, medical aſſiſtance cannot be immediately obtained; and, when it is further conſidered, how very general is the danger and alarming its conſequences, it may be deemed an act of humanity to recommend this valuable and truly uſeful little inſtrument, as a conſtant appendage to the ſideboard of every private family.

It is made of five or ſix threads of catgut, nearly three inches long, twiſted round a wire that paſſes through a flexible tube, and protrudes about two inches and a half beyond its extremity. One end of the twiſt is fixed to the end of the tube *a*, and the other ſomewhat tapered to *that* of the wire *b*.

In its contracted form, (fig. 4) it is to be introduced ſo far into the throat, that all the catgut may be ſuppoſed to have got beyond or below the bone or other ſubſtance to be extracted. The ring at the end of the handle *d*, and outſide of the mouth, is then to be drawn upwards about an inch, by which the catgut threads are extended, as in fig. 5; and thus withdrawing the inſtrument, the bone, &c. will be extracted with it.

Fig. 4. Shews the inſtrument complete, and in the form in which it is to be introduced into the throat. A. The flexible tube. B. C. The twiſted catgut. D. The ring at the end of the wire, on which the catgut threads are fixed.

Fig. 5. The inſtrument when introduced into the throat, with the threads extended under the ſubſtance to be extracted.

The above inſtrument, with Mr. Cruikſhank's admirable contrivance for extracting pieces of money, &c. and the ſponge probang, arranged in a compact caſe, are at all times to be met with at Savigny's.

Fig. 6. The improved ſeton needle.

Fig. 7. The ſame, with the tape of elaſtic gum attached.

---

### Mr. Jardine's Letter.

Sir, *London*, 10*th of May*, 1802.

The liberal and diſintereſted manner in which you have aſſiſted me, by executing the various inſtruments agreeable to the enclofed defigns, juftly entitles you, as a tradefman, to what future benefits may be derived from the fale of others, formed from the fame defigns.

You have, therefore, my permiffion to make from the faid defigns, as many of the inftruments, as may be required, and alfo to publifh, in any manner you may think moft proper, the defcriptions of the fame, with which I have furnifhed you.

In the hope (for your fake) that the demands may be confiderable,

I remain, Sir,

Your obliged and moft obedient fervant,

W. JARDINE.

Mr. JOHN SAVIGNY,

*King Street, Covent Garden.*

## Figures and Tables

**Fig. 7 Fig. 6 Fig. 1 Fig. 2 Fig. 3 Fig. 4 Fig. 5 f1:**
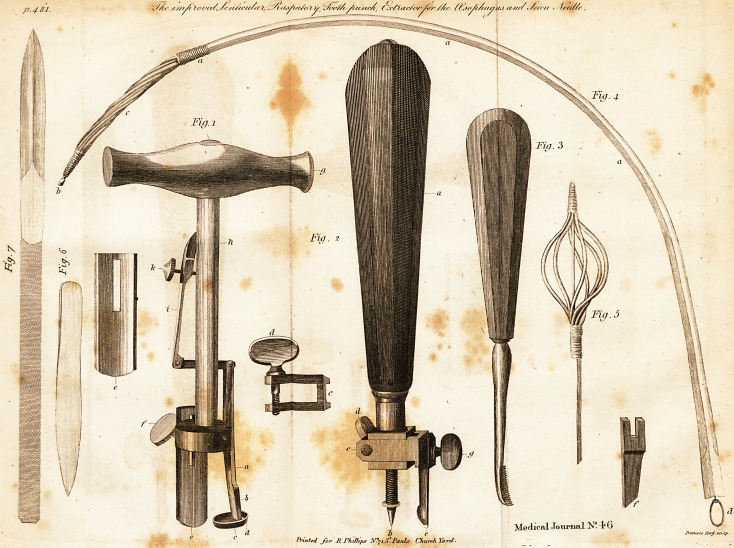


**Fig. 1 f2:**